# The course of African swine fever in Romanian backyard holdings – A case report

**DOI:** 10.1002/vms3.592

**Published:** 2021-08-11

**Authors:** Felix Ardelean, Anja Globig, Alin I. Gârdan Năvălici, Sandra Blome, Klaas Dietze, Klaus Depner, Laura Zani

**Affiliations:** ^1^ County Sanitary Veterinary Health and Food Safety Directorate Satu Mare România; ^2^ Friedrich‐Loeffler‐Institut Federal Research Institute for Animal Health Germany

**Keywords:** African swine fever, backyard sector, disease control, disease spread, outbreak investigation

## Abstract

African swine fever (ASF) was diagnosed for the first time in Romania in a backyard holding in Satu‐Mare County in July 2017. Since then, more than 3800 outbreaks occurred in the entire country. Disease control strategies in the backyard sector rely almost exclusively on reactive measures implemented upon appearance of clinical signs and laboratory confirmation of ASF. In our descriptive study, infection course and outbreak investigation data of 56 affected backyard holdings in Satu‐Mare County has been investigated. Early disease detection based on clinical signs appeared to be efficient. In the majority of outbreaks, ASF was detected within the first 2 weeks after the estimated virus introduction. A clinical phase of 2–8 days was observed before pigs either succumbed to the disease or control measures were implemented on affected farms. A moderate on‐farm transmissibility of ASF virus between pigs was observed. Four clusters of outbreaks were identified indicating virus perpetuation and transmission from farm to farm. To suspend infection chains, rapid intervention by isolating affected farms combined with effective biosecurity measures is required. However, due to the backyard peculiarities, quick and effective implementation of control measures has shown to be rather difficult.

## Graphical abstract

In our case report, infection course and outbreak investigation data of 56 affected backyard holdings in Satu‐Mare County has been investigated. Early disease detection based on clinical signs appeared to be efficient. In the majority of outbreaks, ASF was detected within the first two weeks after the estimated virus introduction.

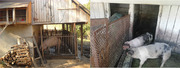

## 1 INTRODUCTION

African swine fever (ASF) is one of the most important viral diseases in domestic pigs and wild boar, causing huge losses in the commercial pig sector as well as affecting livelihood of small‐scale pig holders in rural areas (Sánchez‐Cordón et al., 2018). ASF viruses (ASFV) belonging to the *Asfarviridae* family cause predominantly an acute disease in pigs characterised by high fever, severe but unspecific clinical signs such as inappetence and somnolence, haemorrhages and almost 100% case fatality rate (Penrith et al., 2019; Pikalo et al., 2019).

The genotype II virus circulating in parts of Europe and Asia entered Romania in July 2017 (OIE GF‐TADs Expert Mission Report, 2017). Subsequently, the Romanian domestic pig sector was affected with more than 3800 outbreaks from 2017 to 2020. The first detected outbreak was notified in a backyard holding with four pigs in Satu‐Mare County, located in the North‐West of Romania, bordering Ukraine and Hungary (Boklund et al., 2020). From 2017 to 2019, the county notified 57 outbreaks, all but one in backyard holdings (Table 1).

**TABLE 1:** Overview of ASF outbreaks in domestic pig holdings in Romania and in Satu‐Mare County

		Outbreaks in Satu‐Mare County	
Year	Outbreaks in Romania	Backyard holdings	Commercial farms
2017	2	2	0
2018	1164	20	0
2019	1728	34	1

Rearing pigs in backyards is part of the traditional living in rural areas in Romania and a significant component of agricultural practices. It represents an important if not the only source of meat supply for the rural community and generates valuable cash income (World Bank, 2007). Backyard pigs are mostly slaughtered at home, usually before Christmas (Relun et al., 2016) or whenever new meat supplies are needed.

Despite the low number of animals per farm, and the low relevance on global trade volumes, backyard holdings can play an important role in the local dynamics of ASF epizootics (Zani et al., 2019) and therefore impact the disease status of a country. A general feature observed in backyard holdings is insufficient biosecurity to prevent introduction and spread of ASF. Pigs in backyard holdings are often fed with kitchen leftovers, cereals and fresh grass. This kind of feed is prone to be contaminated with ASFV and thereby representing a high risk for disease introduction (Bellini et al., 2016). Although swill feeding is legally banned, in practice it is difficult to control (Boklund et al., 2020).

In experimental studies with ASFV genotype II strains, the average time between infection and death has shown to be around 10 days, rather independent from the virus dose applied (Pietschmann et al., 2015). This period includes an incubation time of around 5 days and the following clinical phase when pigs show clinical signs and shed virus. Particularly in the early clinical phase, clinical signs are rather unspecific and often mistaken for other infectious diseases. For effective disease control, the time between occurrence of clinical signs and isolation of the affected holdings should be as short as possible. The timely isolation of the affected holding and culling of animals is likely to be a crucial factor for reducing virus transmission and secondary infections. The earlier interventions are taken the lower is the probability of virus transmission to other farms.

So far, there are not many reports published, dealing with ASF in backyard holdings outside of Africa (Boklund et al., 2020; Zani et al., 2019). Our study aims to describe main findings (morbidity, mortality and laboratory data) of ASF outbreaks in backyard holdings in Satu‐Mare County. The data contributes to a better understanding of ASF in backyard settings, particularly regarding the course of the disease and the transmission patterns within and between holdings. This knowledge could help to improve regional strategies addressing ASF control in the backyard sector.

## 2 METHODS AND DEFINITIONS

### 2.1 Methods

In the presented study, data from 56 ASF outbreaks from 2017 to 2019 in backyard holdings of Satu‐Mare county have been analysed. The region of Satu‐Mare was selected for this study due to the fact that it was the first county where ASF occurred in Romania. The data has been obtained during routine outbreak investigation and has been analysed retrospectively. Satu‐Mare County with an area of 4418 km^2^ has around 15,000 backyard holdings with about 68,000 pigs, on average four pigs per holding, depending on the season (OIE GF‐TADs Expert Mission Report, 2017). Initial ASF suspicion was usually raised by the owner who informed the local veterinarian or the veterinary technician of the village. Subsequently, local veterinarians informed competent authorities to confirm or rule out the disease and implement required measures.

Outbreak investigations were conducted by local veterinary authorities as required by EU legislation (EC, 2016). To assess possible virus introduction routes, a hypothesis‐based approach was used as described by Lamberga et al. (2018). The respective holdings were inspected by local veterinary authorities and data was collected from farmers and private veterinarians by interviews. Clinical and laboratory findings, farm settings, biosecurity conditions, animal movements, feeding procedures and human movements were analysed along with morbidity and mortality data. According to the obtained information the most likely sources of virus introduction were ranked according their probability as described in Zani et al. (2019).

For disease confirmation, whole blood samples were tested for ASFV genome and serum samples were tested for ASF‐specific antibodies in the regional laboratory of Satu‐Mare County. Samples were obtained from pigs showing clinical signs indicating an ASF infection. For the detection of viral genome, a commercial qPCR kit (BioRad) was carried out according to manufacturers’ instructions. For antibody detection, a commercial ELISA kit (ID SCREEN African swine fever virus INDIRECT, IDvet) was carried out according to manufacturers’ instructions.

The duration of the clinical phase of individual pigs was estimated by analysing farmer reports. Loss of appetite, skin haemorrhages or increased body temperature were considered as indicative for ASF if the disease could be confirmed later by laboratory testing. For example, if the farmer reported that one pig stopped eating 4 days before it died, we defined the duration of the clinical phase to be 4 days. Based on this data set, the course of the disease was reconstructed to assess timelines for each farm and define disease cluster.

### 2.2 Definitions

**FIGURE 1:**
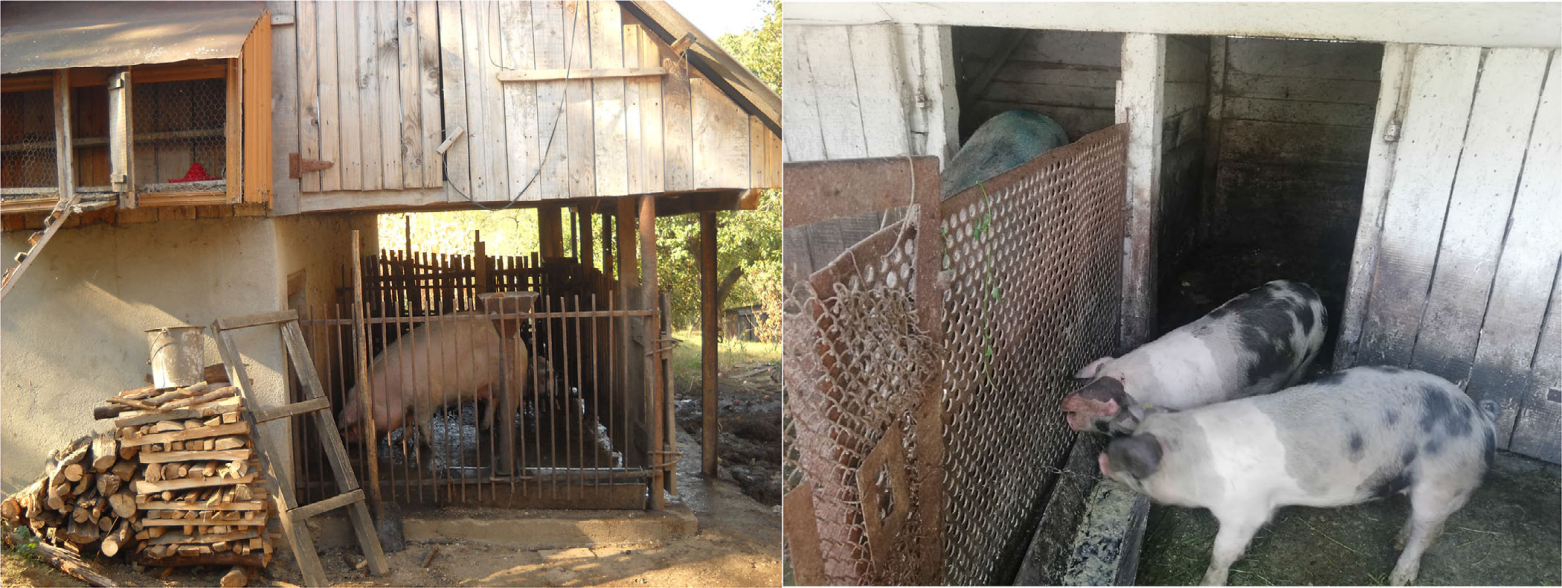
Common setting of a traditional Romanian backyard holding (Pictures: Felix Ardelean)

Clinical phase: Time from the first reported appearance of clinical signs in pigs on the farm until death or culling.
Backyard holding: The designation ‘backyard holding’ stands for a quite heterogeneous family‐run small‐scale pig farming system. Common features are non‐professional or semi‐professional management and low biosecurity. A backyard holding in Satu‐Mare County consists of the residential building and a farmyard with one or more small stables. Usually, pigs are permanently confined without contact to pigs of other farms or wild boar. Outdoor keeping or free ranging of pigs is uncommon. Pigs kept in separate stable buildings do not have direct contact. However, indirect contact via shared feeding and cleaning equipment cannot be excluded. One stable can be divided in several pens where the pigs cannot mingle but have reduced direct contact (Figure 1). As this setting might impact within‐farm spread, pens per farm are displayed in Figure 3d.

## 3 RESULTS

**FIGURE 2:**
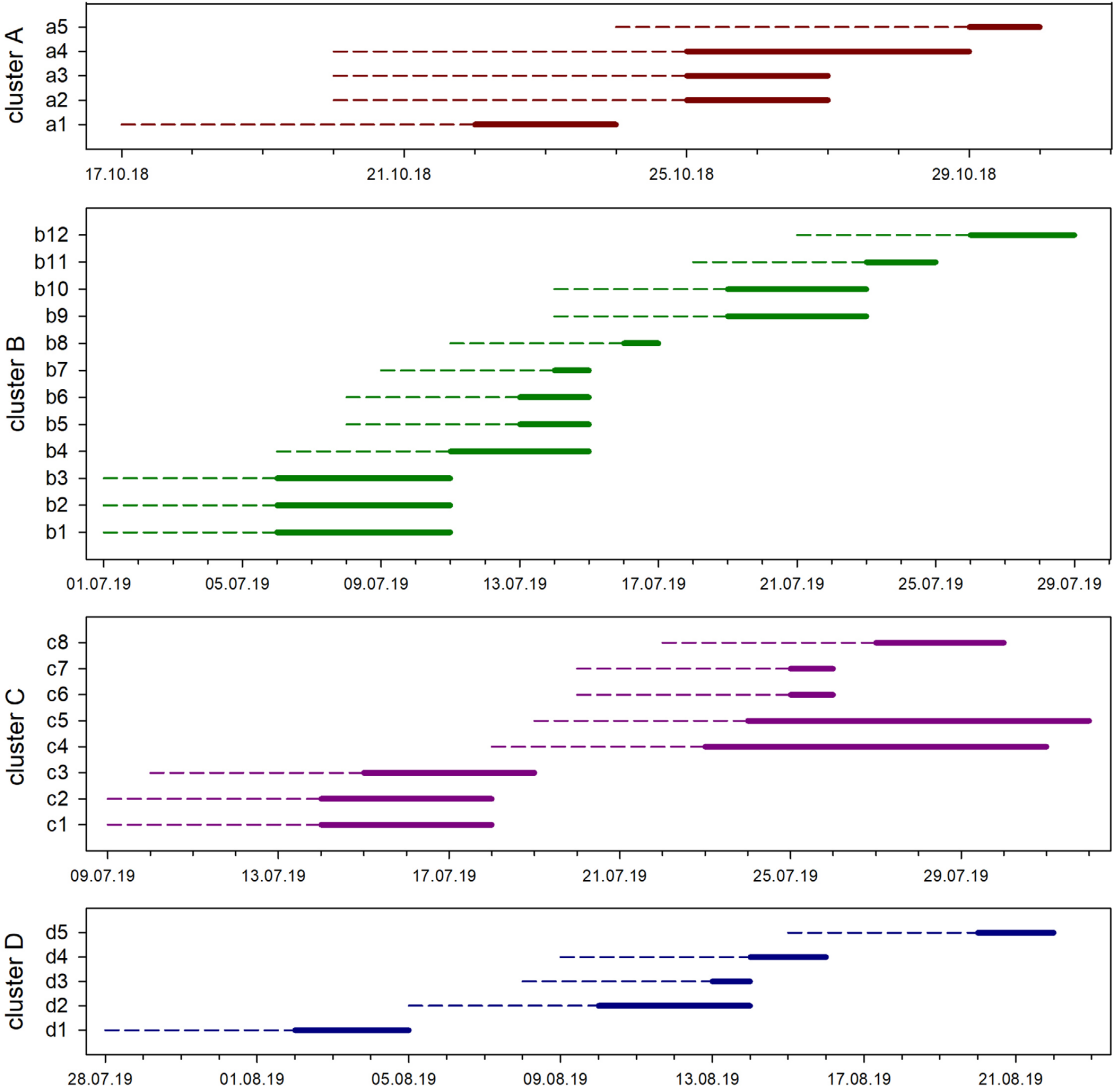
Timelines of clusters. The lines indicate the estimated incubation period of 5 days (short‐dashed line) and the observed clinical phase (solid line)

From the very first case in July 2017 until 2019, 56 ASF outbreaks were notified in backyard holdings in Satu‐Mare County (Table 1). Four clusters (A, B, C, D) including 30 out of 56 outbreaks were defined due to their geographic and temporal proximity and by analysing outbreak investigation data (Figure 2, Supporting Information 1–3). For 26 outbreaks no cluster could be defined.

**FIGURE 3:**
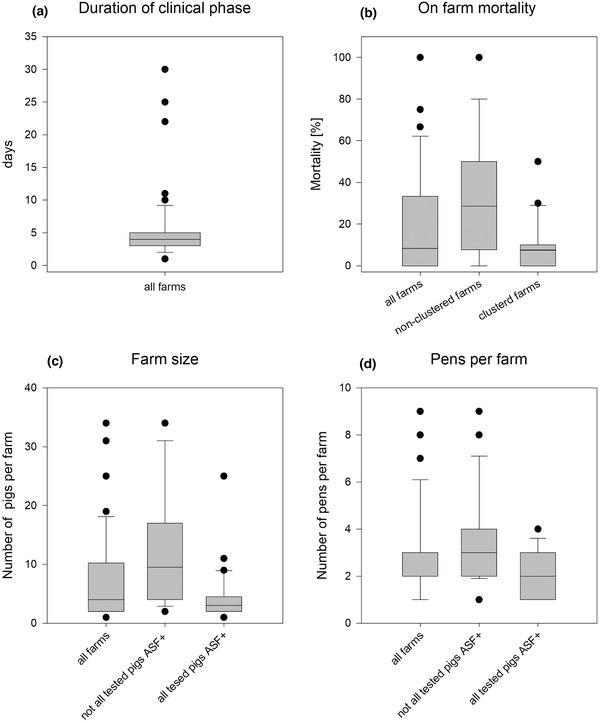
Epidemiological findings and laboratory results related to outbreak farm characteristics

**FIGURE 4:**
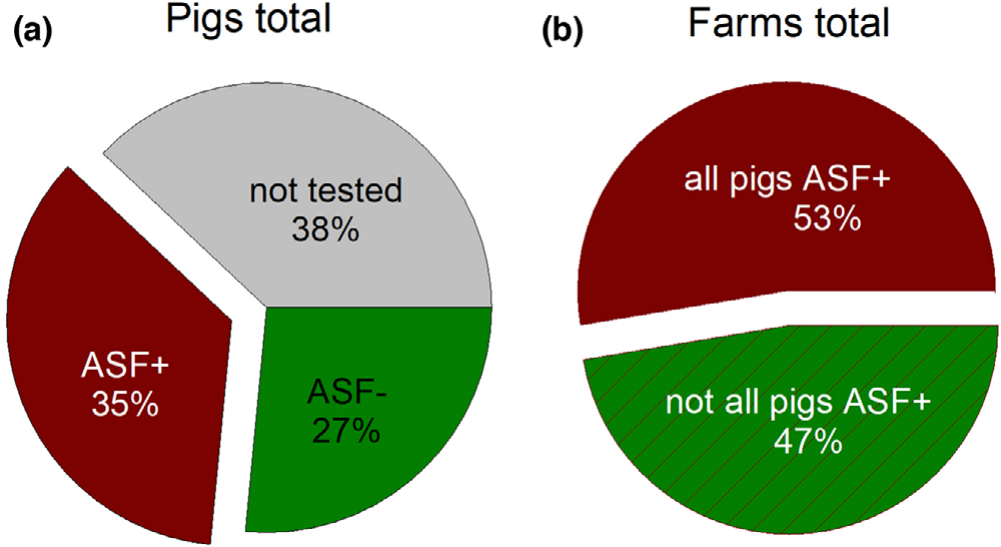
Overview of laboratory results (qPCR). The pie charts show the proportion of negative/positive/not tested pigs (a) and the proportion of farms where all pigs have been tested positive for ASF or where at least one pigs was tested negative for ASF (b)

The number of pigs kept in ASF affected backyard holdings varied from one to 31 (Figure 3c). Twelve holdings had only one pen with up to nine pigs, while the others had two or more pens in up to four stable buildings (Figure 3d). In total, 445 pigs were kept in the affected backyards, 276 were tested for ASF virus genome and 158 were found positive (Figure 4a). In all holdings, pigs with clinical signs were present at the time of disease confirmation. In two farms, pigs with antibodies were detected. In 30 holdings (53%), all tested pigs were PCR positive (Figure 4b). Mortality was reported in 34 backyards while in 23 holdings ASF was confirmed before pigs died. The mortality was higher in farms that were regarded to be not part of a disease cluster compared to clustered farms (Figure 3b).

The shortest clinical phase was estimated to be 2 days (seven holdings) and the longest 31 days (one holding). On 49 backyard holdings (86%), the clinical phase was estimated to be less than 10 days (Figures 2 and Figure 3a).

Regarding the possible virus introduction routes into the farms, contaminated swill was ranked as the most likely source of infection based on the results of the outbreak investigation. Within farms, direct or indirect contact between pigs was identified as the most probable reason for virus spread.

## 4 DISCUSSION

Basically, three animal health policy pillars exist for controlling the spread of a (notifiable) infectious disease like ASF, where no vaccine and treatment are available: (i) timely identification of infected animals and holdings, (ii) immediate isolation of the affected holding and culling of animals and (iii) identification of contact holdings (Fraser et al., 2004). These measures rely on early reporting of disease suspicion followed by rapid laboratory confirmation. In case of ASFV Genotype 2, the clinical phase starts usually after an incubation period of about 3–5 days (Gabriel et al., 2011) which would be the earliest time point during the ASF infection when the owner might suspect that one of his pigs is sick. In the backyard context, detecting suspect animals depends nearly exclusively on the pig owner, which may or may not report suspect cases. Farmers of backyard holdings are often not familiar with clinical signs of ASF or with its specific epidemiological features. However, due to close and frequent contact with their animals they instantly notice if a pig behaves different. This observation raises concern about the health status and may ideally trigger the report to the local veterinarian.

Backyard holdings in general are, due to their small size and few animals, in favour for early detection of ASF since sick and dead animals are usually spotted relatively early during an infection period. In large commercial farms, ASFV might circulate for several weeks before it causes a substantial increase in mortality and the disease is notified (Bech‐Nielsen et al., 1995; Dione et al., 2017; Lamberga et al., 2018). In large farms, the very first animals falling sick and dying from ASF might be overlooked if there is no enhanced passive surveillance system in place targeting dead and sick animals with a specific follow‐up (Lamberga et al., 2018). In the majority (86%) of the farms of our study, the estimated clinical phase lasted 2–6 days before measures (isolation of the backyard, culling of animals) were implemented. Hence, it can be hypothesised that on these farms ASF was detected towards the end of the first infection period of around 10 days (Gabriel et al., 2011). This hypothesis is supported by the fact that in 40% of the backyard holdings the disease was discovered before pigs succumbed to the disease.

On two backyard holdings, seropositive animals were found, indicating an infection period of more than 10 days (Gabriel et al., 2011). Generally, ASFV‐specific antibodies have been rarely reported from European countries affected with ASFV Genotype 2 as pigs mostly succumb to the disease before seroconversion. In other backyard holdings, clinical signs were noticed in different animals over a period of up to 31 days but no suspicion was raised. The observed variations can be explained with differences between holdings, unspecific clinical signs as well as with the subjectivity in reporting clinical observations.

In 47% of the holdings, not all tested pigs were ASF positive. It can be hypothesised that in the respective holdings, only one or few pigs got initially infected and that the virus did not spread to other pigs within one infection period. These findings are in line with literature (Chenais et al., 2019; Zani et al., 2019) and contribute to the hypothesis that ASF is moderately contagious. Nevertheless, in 53% of the backyard holdings, all tested pigs were ASF positive. This infection pattern was mainly seen on farms with few pigs. It can be hypothesised that in those holdings all tested pigs got infected at once. In 24 holdings, not all pigs were tested making it difficult to interpret the data set accordingly. The reason for the incomplete set of samples is the fact that the data was generated during routine outbreak investigation where authorities sampled mainly pigs showing clinical signs.

Swill is considered to be generally the most likely source of virus introduction in the backyard sector (Costard et al., 2015; Heilmann et al., 2020). In experimental studies, it was proven that the oral infectious dose may have an impact on the infection rate. To efficiently infect healthy pigs by the oral route, more than 10,000 infectious units of ASFV are needed (McVicar, 1984). However, with much lower doses, weaker animals will pick up the infection and a scattered infection pattern is observed in the herd (Pietschmann et al., 2015). Even with high infectious doses, some animals may not get infected and contagiousness can be moderate (Gabriel et al., 2011; Zani et al., 2018). Nevertheless, it could be proven that liquids (e.g. contaminated water) containing low doses of virus are more infectious than contaminated forages (Niederwerder et al., 2019).

In our study, we observed a delayed disease transmission if pigs were kept in separate pens and different stables. This is in line with experimental studies, where it could be shown that an infectious pig would infect on average 5.0 animals within one pen and 2.7 animals between pens. The within‐pen transmission might be facilitated by blood contact (high virus dose). Contaminated materials, for example contaminated stable equipment or clothes of workers might contribute to the in‐between pen transmission (Guinat et al., 2016). A similar observation was made in an outbreak in a backyard holding in Bulgaria (Zani et al., 2019).

We identified four clusters of outbreaks (A, B, C, D). Due to their proximity in time and space, we assumed that the outbreaks within a cluster were epidemiologically linked. However, clear proofs for possible links between the farms were not found. Timelines of clustered outbreaks suggest that in some cases simultaneous infections of farms occurred leading then to secondary outbreaks. It can be assumed that the virus leaped from one backyard holding to the next due to insufficient biosecurity measures, for example frequent movements of people and shared equipment. The observed mortality in clustered outbreaks was found to be lower than in non‐clustered outbreaks. This can be explained by the fact that after disease confirmation neighbouring farms were checked timely leading to earlier disease detection.

To facilitate early detection and to avoid secondary spreading, it is crucial that after an outbreak, all neighbouring backyard holdings are inspected immediately for the presence of ASF. This measure should be repeated every week, at least for 1 month after the last outbreak. In particular, the presence of clinical signs and mortality should be checked. If suspect or dead animals are found, samples should be taken and tested for ASFV. Additionally, incentives for backyard farmers that report timely sick and dead animals could facilitate early detection. The success of such a reactive strategy is mainly depending on the rapid intervention by isolating affected farms combined with effective biosecurity measures.

## CONFLICT OF INTEREST

The authors declare that the research was conducted in the absence of any commercial or financial relationships that could be construed as a potential conflict of interest.

## AUTHOR CONTRIBUTIONS

Felix Ardelean: Conceptualisation, data curation, formal analysis, investigation, writing‐review and editing. Anja Globig: Conceptualisation, formal analysis, methodology and writing‐original draft. Alin I. Gârdan Năvălici: Conceptualisation, formal analysis, writing‐review and editing. Sandra Blome: Validation, writing‐review and editing. Klaus Depner: Conceptualisation, supervision and writing‐original draft. Laura Zani: Conceptualisation, formal analysis, validation and writing‐original draft.

### PEER REVIEW HISTORY

The peer review history for this article is available at https://publons.com/publon/10.1002/vms3.592

## Supporting information

Supporting Information

Supporting Information

## Data Availability

The original contributions presented in the study are included in the article and supplementary material; further inquiries can be directed to the corresponding author.
