# Supplementary figures and images for: The course of African swine fever in Romanian backyard holdings – A case report
Source: Vet Med Sci. 2021 Aug 11;7(6):2273–9. doi: 10.1002/vms3.592 (PMC8604127; doi:10.1002/vms3.592)

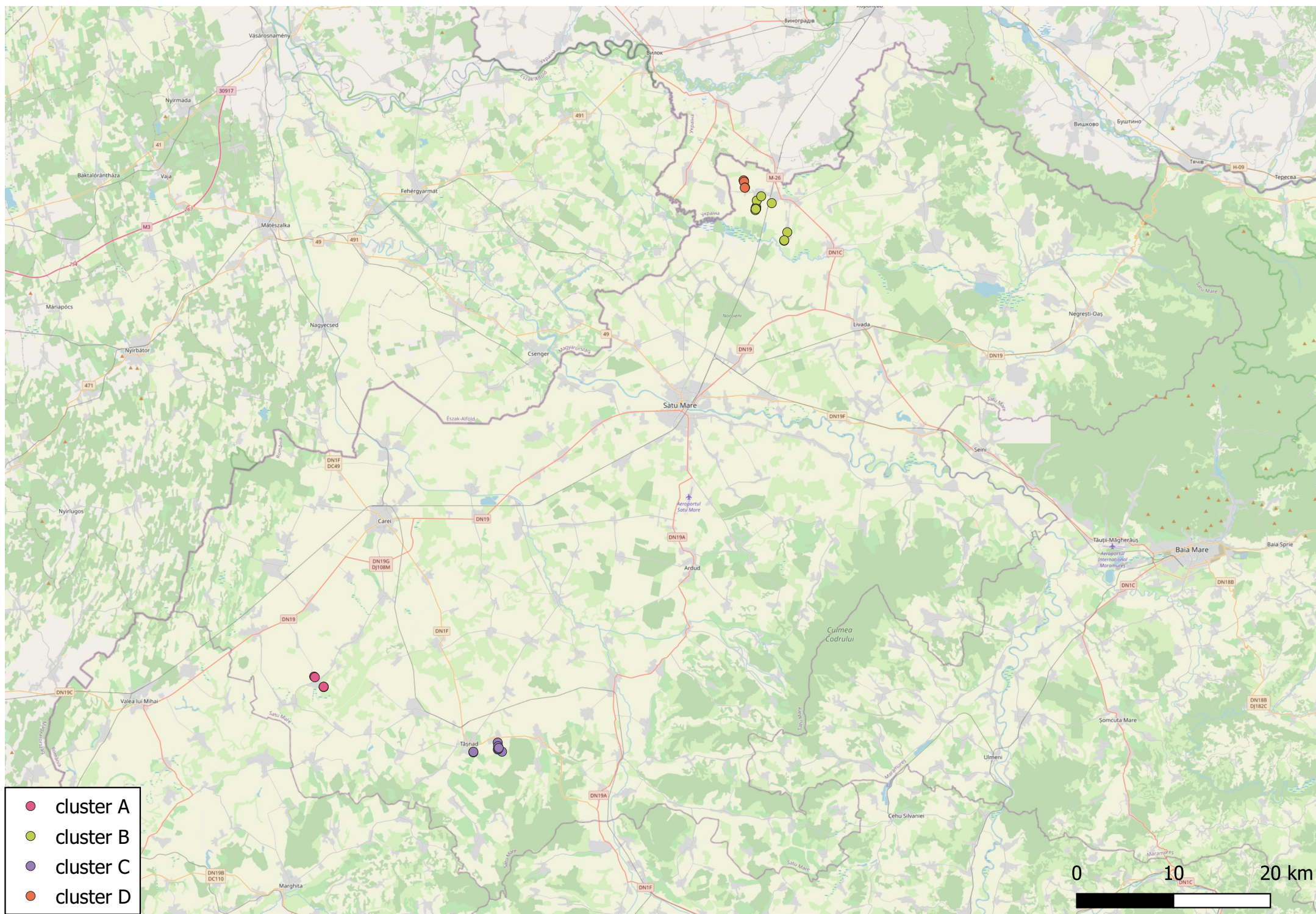

Supplement: Supplementary file 1 — Supporting Information [file VMS3-7-2273-s002.pdf]

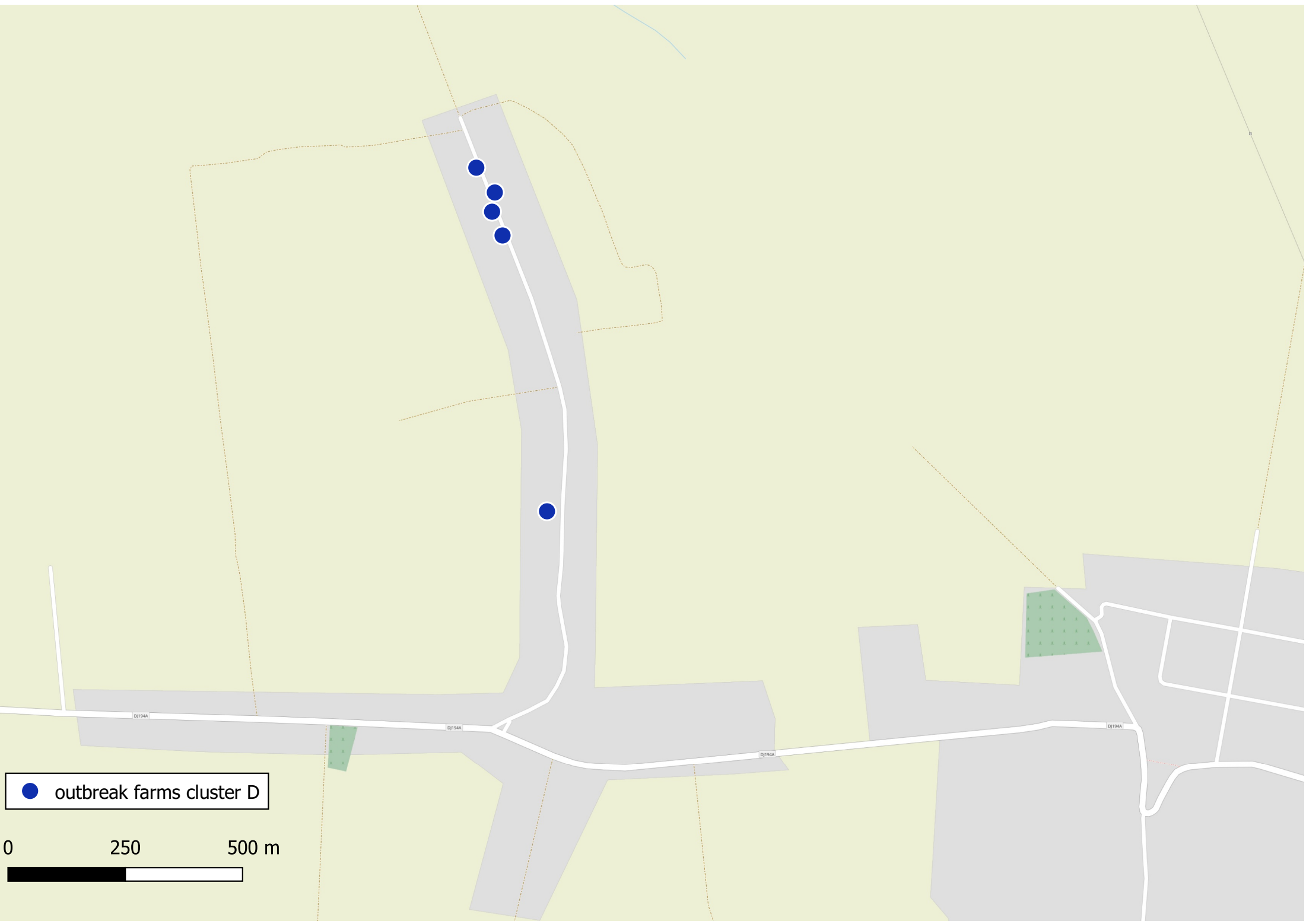

● outbreak farms cluster D

0 250 500 m

Supplement: Supplementary file 2 — Supporting Information [file VMS3-7-2273-s001.pdf]
